# *In silico*, *in vitro*, X-ray crystallography, and integrated strategies for discovering spermidine synthase inhibitors for Chagas disease

**DOI:** 10.1038/s41598-017-06411-9

**Published:** 2017-07-27

**Authors:** Ryunosuke Yoshino, Nobuaki Yasuo, Yohsuke Hagiwara, Takashi Ishida, Daniel Ken Inaoka, Yasushi Amano, Yukihiro Tateishi, Kazuki Ohno, Ichiji Namatame, Tatsuya Niimi, Masaya Orita, Kiyoshi Kita, Yutaka Akiyama, Masakazu Sekijima

**Affiliations:** 1 Advanced Computational Drug Discovery Unit, Institute of Innovative Research, Tokyo Institute of Technology, 4259-J3-23, Nagatsuta-cho, Midori-ku, Yokohama 226-8501 Japan; 2 Education Academy of Computational Life Sciences (ACLS), Tokyo Institute of Technology, Yokohama, 226-8501 Japan; 3 Global Scientific Information and Computing Center, Tokyo Institute of Technology, 2-12-1 Ookayama, Meguro-ku, Tokyo 152-8550 Japan; 4 Graduate School of Agricultural and Life Sciences, The University of Tokyo, Bunkyo-ku, Tokyo 113-8657 Japan; 5 Medicinal Chemistry Research Labs, Drug Discovery Research, Astellas Pharma Inc, 21 Miyukigaoka, Tsukuba, Ibaraki 305-8585 Japan; 6 Department of Computer Science, Graduate School of Information Science and Engineering, Tokyo Institute of Technology, Meguro-ku, Tokyo 152-8550 Japan; 7 Department of Biomedical Chemistry, Graduate School of Medicine, The University of Tokyo, Bunkyo-ku, Tokyo 113–0033 Japan; 8 School of Tropical Medicine and Global Health, Nagasaki University, Sakamoto, Nagasaki 852–8523 Japan; 9 Catalyst Inc., Risona Kudan Building 5F KS Floor, 1-5-6 Kudan Minami, Chiyoda-ku, Tokyo 102-0074 Japan

## Abstract

Chagas disease results from infection by *Trypanosoma cruzi* and is a neglected tropical disease (NTD). Although some treatment drugs are available, their use is associated with severe problems, including adverse effects and limited effectiveness during the chronic disease phase. To develop a novel anti-Chagas drug, we virtually screened 4.8 million small molecules against spermidine synthase (SpdSyn) as the target protein using our super computer “TSUBAME2.5” and conducted *in vitro* enzyme assays to determine the half-maximal inhibitory concentration values. We identified four hit compounds that inhibit *T*. *cruzi* SpdSyn (TcSpdSyn) by *in silico* and *in vitro* screening. We also determined the TcSpdSyn–hit compound complex structure using X-ray crystallography, which shows that the hit compound binds to the putrescine-binding site and interacts with Asp171 through a salt bridge.

## Introduction

Chagas disease results from infection by *Trypanosoma cruzi* and is a neglected tropical disease (NTD)^1^. This disease is endemic to approximately 20 countries, including the southern United States and parts of Latin America^1–3^, and the World Health Organization (WHO) estimates that as many as 10–13 million people are chronically infected with this disease^3, 4^. Moreover, approximately 90 million people are exposed to the risk of infection annually, and 21,000 deaths are reported each year^3–5^. Accordingly, Chagas disease remains a serious vector-borne infection^6^.

The infection routes of *T*. *cruzi* primarily occur through contact with the feces of blood-sucking *Triatominae* and blood transfusion^7^. Chagas disease is classified into acute and chronic phases^8, 9^. The acute phase is characterized by high blood parasitemia and fever. In some cases, myocarditis or meningoencephalitis is caused by parasitization in the cardiac muscle or the brain during acute infection. The chronic phase results from an untreated acute phase, and approximately 20–30% of chronical infections are symptomatic^3, 4, 8^.

Currently, two drugs developed during the 1960s—nifurtimox and benznidazole—are used to combat *T*. *cruzi* infections. These drugs are effective during both the acute and early chronic phases. However, their use is associated with serious side effects, and they exhibit limited efficacy during the chronic phase of Chagas disease^7, 8, 10^. Therefore, the development of new anti-Chagas drugs is required.

To this end, we focused on spermidine synthase (SpdSyn) as the target protein, as sourced from the iNTRODB system^11^. Detailed information regarding the search method is presented in Supplementary Figure 1. The in-house web-system iNTRODB facilitates the selection of drug target proteins for NTDs, particularly for trypanosomiasis. This system provides information on trypanosomal proteins with useful annotations, including the protein structure from the Protein Data Bank (PDB) and the protein inhibitors from ChEMBL. Using this system, a user can rank the records by specific features and, thereby, identify possible candidates. When selecting SpdSyn as the target, we focused on features such as RNA interference target sequencing (RIT-seq) screening results and protein crystal structure availability. The RIT-seq screening results are taken from TriTrypDB^12^, which is integrated into iNTRODB. The screen shows that SpdSyn is important for survival in a related species (i.e., *Trypanosoma brucei*). Additionally, a structural examination of the protein by X-ray crystallography, which is useful in structure-based drug design (SBDD), is feasible since several successful structure determinations have been reported^13^. These two properties suggest that the inhibition of SpdSyn is a probable mechanism of action (MOA) for the treatment of Chagas disease and that the SBDD approach for inhibitor design is viable.

The polyamine biosynthesis pathway is an attractive target for the development of new antiparasitic agents. Polyamines adopt various roles in trypanothione biosynthesis and stabilization and replication of the DNA structure^14–17^. Genetic experimental data have shown that SpdSyn is essential for maintaining the viability of a parasite during *T*. *brucei* gene knockout or RNA interference (RNAi) studies^18–23^. This protein is classified as an enzyme that participates in the polyamine synthesis pathway using decarboxylated *S*-adenosylmethionine (dcSAM) as an aminopropyl donor. The enzyme catalyzes the transfer of polyamine from dcSAM to putrescine giving spermidine^24–26^. Importantly, the SpdSyn gene is conserved among protozoan parasites, such as *T*. *cruzi* and *Leishmania* spp. (TcSpdSyn: PDB ID code: 3BWB and 3BWC, *Leishmania major* SpdSyn: UniProt ID Q95Z84). Supplementary Figure 2 shows good alignment of a multiple amino acid sequences of trypanosomatid SpdSyns, with high identity, when compared to human SpdSyn, leads to a lower identity (67%, 60%). Although the polyamine biosynthesis pathway has been identified as a potential drug target pathway in *T*. *brucei*, *Leishmania* spp., and *T*. *cruzi*, the druggability of SpdSyn in *T*. *cruzi* remains inconclusive. To determine whether TcSpdSyn is a druggable enzyme, a specific inhibitor is required to test the effect of polyamine biosynthesis inhibition on the parasite using small molecules.

**Figure 1:**
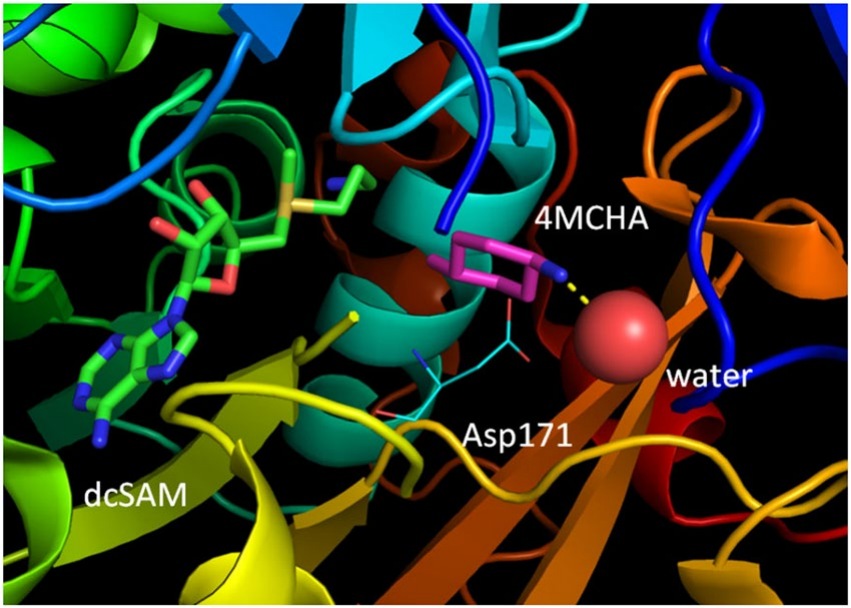
The crystal structure of the TcSpdSyn putrescine-binding site with trans-4-methylcyclohexylamine (4MCHA) (PDB ID code: 4YUW). 4MCHA binds to the putrescine-binding site and interacts with Asp171 through a salt bridge. A water molecule interacts with the 4MCHA amino group.

The crystal structure of the complex of TcSpdSyn and a substrate analogue, trans-4-methylcyclohexylamine (4MCHA), has been determined^27^. Figure 1 shows the binding mode of 4MCHA in the crystal structure (PDB ID code: 4YUW). This ligand interacts with Asp171 through a salt bridge and has been demonstrated to inhibit TcSpdSyn with a half-maximal inhibitory concentration (IC_50_) of 1.7 μM^27^. Asp171 is a part of the gatekeeping loop which is important in substrate binding^28^. Hence, SpdSyn inhibitors could interact with Asp171 to bind in the SpdSyn active site. However, because 4MCHA is too small in comparison with other pharmacologically active compounds and has only one hydrogen bond donor, this ligand would have low specificity, and its optimization as an anti-Chagas drug would be difficult. So, we decided to take a different approach to develop an anti-Chagas drug with optimal properties.

Docking simulation is a representative SBDD technique and is a computational method used to search for drug candidates^29, 30^. This simulation evaluates how well a compound fits into a binding pocket of the target protein by calculating a score function (e.g., the strength of the interactions between the compound and the target protein). Docking simulations can be complemented with data obtained through wet experimental methods to produce a compound hit rate^31^, which is a powerful tool for identifying new inhibitors with different core structures^13^.

The fragment molecular orbital (FMO) method^32^ conducts *ab initio* quantum mechanical calculations on large biomolecules, such as protein–ligand complexes, and is not only useful in identifying important interacting residues but also in determining the binding energy between a ligand and a target protein^32^. This method has proven to be useful in the design of new drugs^33–39^.

In the present study, we performed docking simulations to identify compounds that inhibit TcSpdSyn through binding to the putrescine-binding site. We evaluated the inhibition activity of the compounds selected from the docking simulations using an *in vitro* assay and performed X-ray co-crystallography to determine the TcSpdSyn structure in complex with the hit compounds. Furthermore, we conducted FMO interaction analysis to determine important interactions between TcSpdSyn and the inhibitors.

## Results

### *In silico* screening

**Figure 2:**
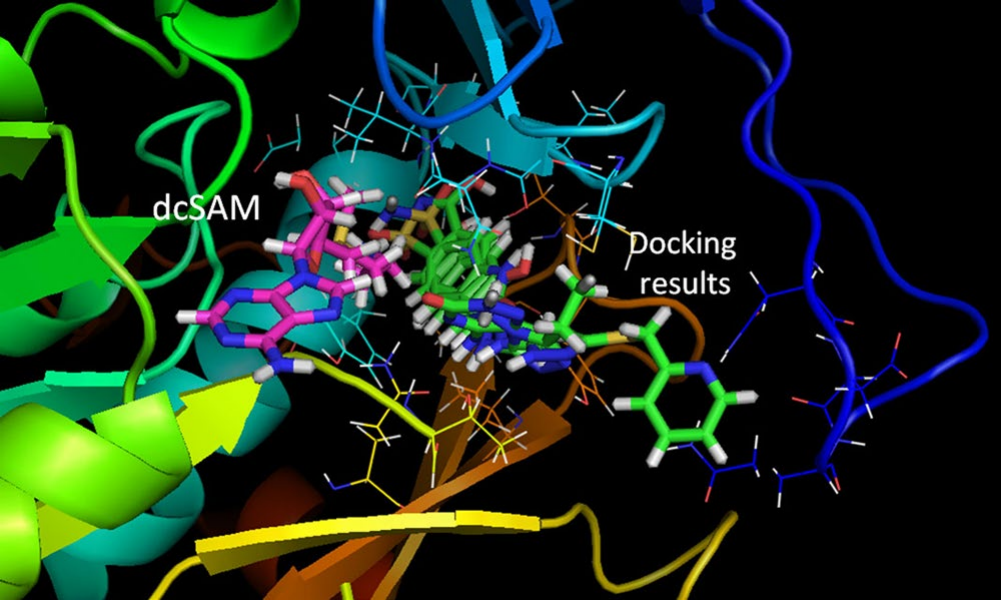
Results of the TcSpdSyn-ligand docking analysis. Docking poses of the top five compounds (green: docking results, pink: dcSAM). This simulation was conducted with the TcSpdSyn X-ray structure (PDB ID code: 3BWC). The docking results show that all compounds bind to the putrescine-binding site and that dcSAM binding in its own site.

To find potential TcSpdSyn inhibitors, we conducted a docking simulation using the TcSpdSyn X-ray structure (PDB ID code: 3BWC). The target site in the docking simulation was set to the putrescine-binding site where the catalytic reaction occurs. The compounds used for the docking simulation were sourced from a database of commercially available compounds. Figure 2 shows the docking poses of the top five potential inhibitors, which had docking scores of −8.36, −7.78, −7.72, −7.71, and −7.63. The docking results varied on the basis of target cavity size and binding compound volume. Therefore, the molecular weights and logP values for 1,000 compounds were lower than the average values of the 4,800,000 drug-like compounds used for docking simulations.

### *In vitro* assay and structure determination

To identify TcSpdSyn inhibitors, we performed an *in vitro* enzyme inhibition assay on 176 compounds sourced from the chemical compound library at Astellas Pharma. The inhibition of TcSpdSyn by the test compounds was measured at a final concentration range between 84.5 and 500 μM, depending on compound availability and solubility. Subsequently, the IC_50_ values of the compounds that showed >40% inhibition were determined. Several compounds with dose-response activity were found, and here, we report four compounds with IC_50_ values between 28 and 124 μM. Table 1 shows the parent chemical structures of active compounds and their IC_50_ values (the fitting curve for the IC_50_ calculation is shown in Supplementary Figure 3). The docking scores of the hit compounds fall within the top 2,000 compounds out of 4,800,000 drug-like compounds. Compounds 1, 3, and 4 contain an amino-alkyl chain connected to an aromatic ring, as is found in putrescine. Therefore, these amino groups should interact with Asp171 through a salt bridge.

**Table 1:** The parent structures of compounds 1–4 and their IC_50_ values.

Compound ID	Parent structure	Compound name	IC_50_ for TcSpdSyn (μM)
1		2-(2-Fluorophenyl)ethanamine	124
2		2-[(4,6-dihydroxy-1,3,5-triazin-2-yl)amino]-4H-1,3-benzothiazin-4-one	28
3		2-(5-Ethoxy-1-ethyl-1H-indol-3-yl)ethanamine	113
4		4-(2-Aminoethyl)-1,2-benzenediol	66
4MCHA		trans-4-methylcyclohexylamine	1.9

These IC50 values were determined from fitting curve in Supplementary Figure 3. Compound name is taken from the following Web site: http://www.chemspider.com/StructureSearch.aspx.

X-ray crystal structures of TcSpdSyn-inhibitor complexes were used to confirm the binding modes of the hit compounds. Although all active compounds were subjected to X-ray crystallography, only the structure of the TcSpdSyn complex with compound 1 was determined. Co-crystallization with the remaining compounds were unsuccessful after repeated attempts, possibly due to alternate binding at a different location, such as the dcSAM binding site.

**Figure 3:**
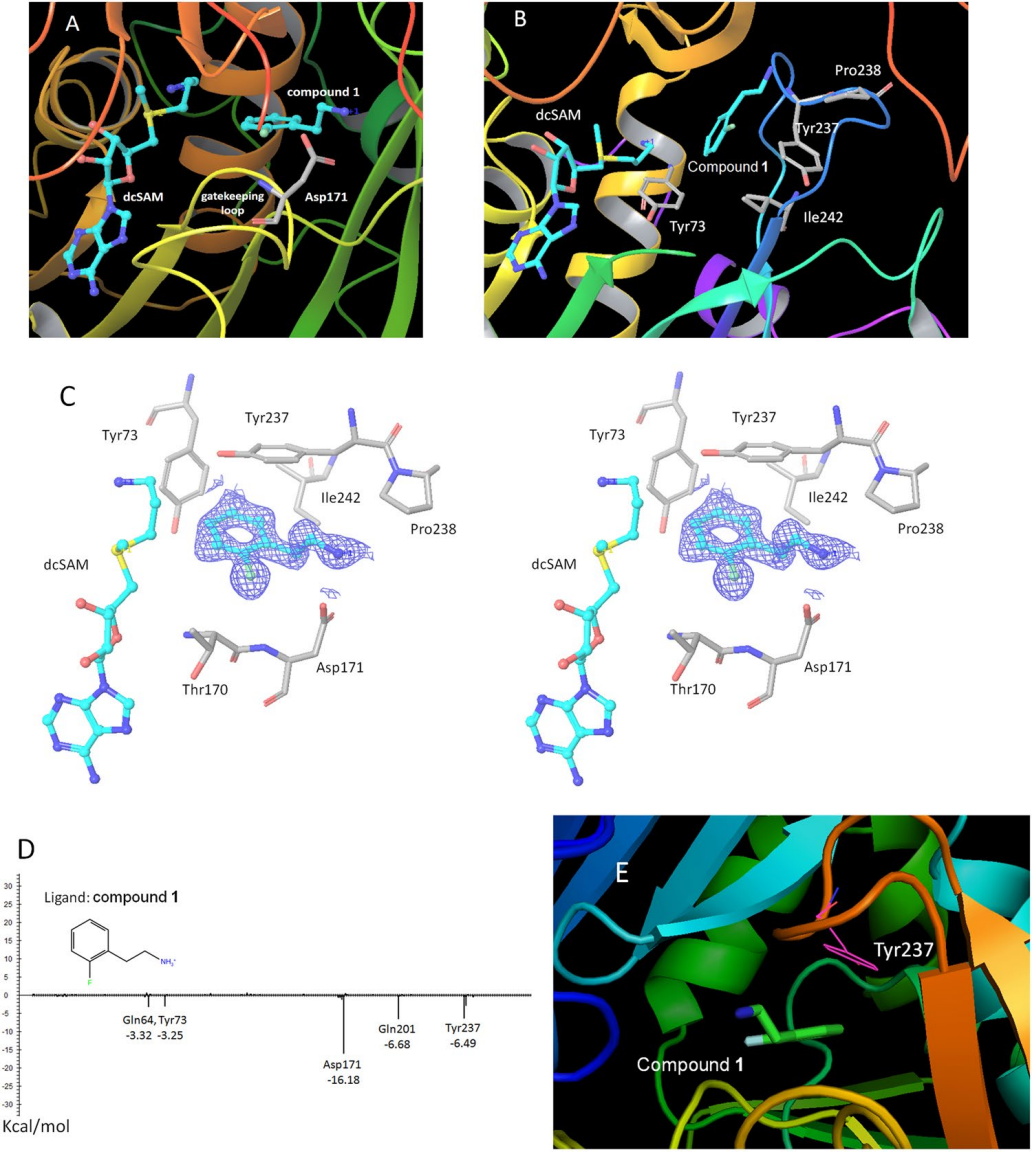
The crystal structure analysis of TcSpdSyn with compound 1. (**A**) The crystal structure of the TcSpdSyn-compound 1 complex. (**B**) Binding form of compound 1 predicted by docking simulation. (**C**) Stereo representation of the TcSpdSyn-compound 1 complex structure. The 2*F*o-*F*c map around compound 1 is shown as a blue mesh (contoured at 1.5σ). (**D**) Interaction energy analysis of compound 1. The vertical axis shows the interaction energy (kcal/mol) between the ligand and amino acid, and the horizontal axis shows the amino acid residue number. (**E**) Interaction between compound 1 and Tyr237.

Figure 3A and C and Table 2 show the crystal structure of TcSpdSyn in complex with Compound 1 (IC_50_ = 124 μM). This data confirms that Compound 1 is bound to the putrescine-binding site. Figure 3B shows that compound 1 is proximally bound to Tyr73, Tyr237, Pro238, and Ile242 in the TcSpdSyn putrescine-binding site (PDB ID code: 3BWC). When comparing the docking pose with the crystal structure, the binding mode obtained by the docking simulation clearly shows there is no correspondence to the observed binding mode in the crystal structure even though, in both binding modes, the position of the aromatic ring of compound 1 is in the polyamine region of dcSAM. In the TcSpdSyn structure that was used for the docking simulation (PDB ID code: 3BWC), the gatekeeping-loop residue Asp171 is disordered. Thus, interaction with Asp171 was unexpected based on the docking simulation.

**Table 2:** Crystallographic analysis statistics of the TcSpdSyn-compound 1 complex.

Data Collection		Refinement	
space group	*P*1	no. of reflections	115798
a, b, c (Å)	44.48, 68.16, 94.46	*R*_work_/*R*_free_	0.188/0.232
α, β, γ (deg)	87.91, 87.18, 80.27	no. of atoms (protein)	9224
resolution (Å)	50.00–1.58 (1.61–1.58)	no. of atoms (water)	583
*R*_merge_	0.055 (0.073)	r.m.s. deviations	
*I/σI*	33.2 (18.0)	bond lengths (Å)	0.023
completeness (%)	81.5 (91.5)	bond angles (deg)	2.403
redundancy	1.9 (1.7)	PDB code	5B1S

The crystal structure indicates that compound 1 interacts with Asp171 by a salt bridge between the amino group of compound 1 and the carboxylate group of Asp171. To determine whether this interaction meets the geometric requirements of the salt bridge, the distance between the amino nitrogen atom of compound 1 and the δ–O atom of Asp171 was measured. Furthermore, by adding the hydrogen atoms to the crystal structure, we measured the bond angles with respect to the nitrogen, the hydrogen atom attached to the nitrogen, and the δ–O atom of Asp171. We found that the distance and bond angle were 2.71 Å and 162.8°, respectively. Because these values meet the geometric requirements of a salt bridge, we concluded that a salt bridge interaction was present. According to the X-ray crystallography result, the ethylene linked amino group facilitates binding to the TcSpdSyn putrescine-binding site. The IDs of inactive compounds are listed in Supplementary Table 2. In the present study, we confirmed the structures of active compounds using mass spectrometry (MS) and nuclear magnetic resonance (NMR).

### Interaction energy analysis

The interaction energy between TcSpdSyn and compound 1 was analyzed by FMO calculation using the crystal structure of TcSpdSyn in complex with compound 1. Figure 3D shows the results of this interaction energy analysis. Compound 1 interacts with Asp171 at the putrescine-binding site (interaction energy value: −16.18 kcal/mol). We also performed FMO calculations using the crystal structure of TcSpdSyn complexed with putrescine and with 4MCHA. The interaction energy values with Asp171 were calculated to be −5.54 and −14.29 kcal/mol, respectively. These significant interaction energy values illustrate interactions with Asp171 and show importance for ligand binding. In addition to the interaction with Asp171, other interactions were confirmed: Gln64 and Tyr73 interact with compound 1 with interaction energy values of −3.32 and −3.25 kcal/mol, respectively. Moreover, Glu201 and Tyr237 interact with compound 1 with interaction energy values of −6.68 and −6.49 kcal/mol, respectively. Figure 3E shows the interaction mode between compound 1 and Tyr237. There appears to stabilization through a π-π interaction between the aromatic ring of compound 1 and the aromatic ring of Tyr237. Thus, the Tyr237 π-electrons play an important role in the binding of compound 1.

### Molecular dynamics (MD) analysis of compound interaction

**Figure 4:**
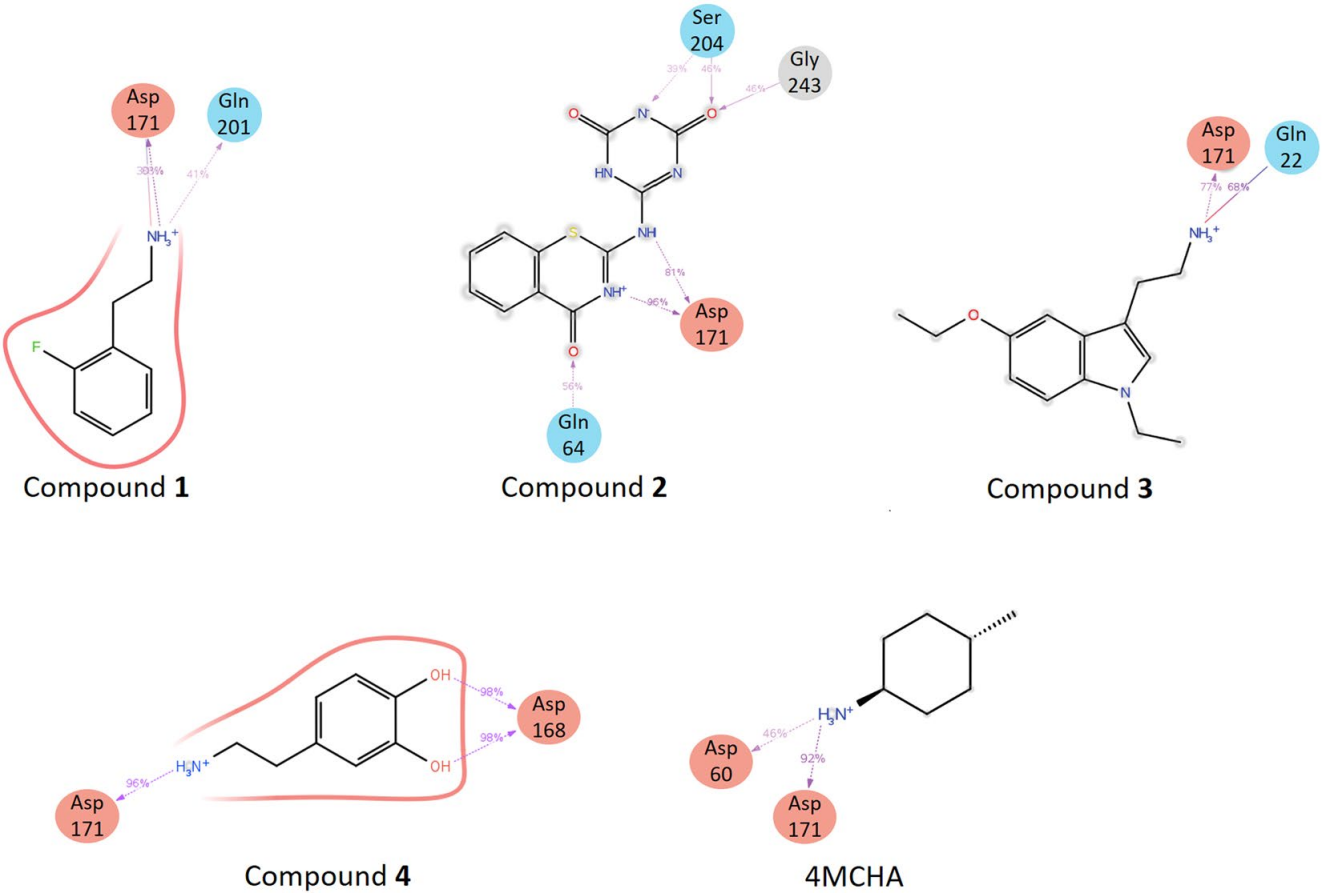
TcSpdSyn-ligand interaction analysis by MD simulation. Schemes of detailed ligand atom interactions with the protein residues are presented. Interactions that occur for more than 30.0% of the simulation time in the selected trajectory (0.00 through 20.00 ns) are shown.

MD simulations were performed for compounds 1–4 and 4MCHA to further investigate their interactions with amino acid residues. For the analysis of compound 1, the complex structure was obtained by X-ray crystallography. For compounds 2–4 the complex structures were obtained by the docking simulation with the compound 1 co-crystal. As a reference, the co-crystal structure of TcSpdSyn and 4MCHA was also subjected to the MD simulation. Figure 4 shows the amino acid residues during the MD simulation. All compounds interact with Asp171 through a salt bridge or hydrogen bond. Moreover, the MD simulation shows that compounds 1–4 interact with other amino acid residues, namely, Gln22, Gln64, Asp168, Gln201, Ser204 and Gly243 suggesting that hit compounds identified in this study have additional interactions that were not predicted by the docking simulations.

## Discussion

Using a virtual screening approach, we performed a docking simulation to identify TcSpdSyn inhibitors from among approximately 4,800,000 drug-like compounds. *In vitro* assays of the compounds selected from the docking simulation were conducted, and the IC_50_ values of four compounds were determined. As a positive control, we also determined IC_50_ of 4MCHA against TcSpdSyn (its value is 1.9 uM and is determined by this study.). Furthermore, to clarify the binding modes of the active compounds, X-ray crystallography was performed. The TcSpdSyn-complex 1 structure was determined, whereas attempts to obtain crystal structures of TcSpdSyn in complex with other compounds were unsuccessful. The obtained crystal structure revealed that compound 1 is bound to the TcSpdSyn putrescine-binding site and forms a salt bridge with Asp171 and a van der Waals interaction with Tyr237. Because these two interactions are also observed in the crystal structure of the TcSpdSyn-4MCHA complex, the binding mode of compound 1 is similar to that of 4MCHA. However, despite the conservation of the binding mode, compound 1 is less active than 4MCHA (124 μM vs. 1.9 μM). We believe that there are two possible explanations for the difference in activity. First, there is a high entropic penalty in binding compound 1 due to the fact that compound 1 contains a flexible aminoalkyl side chain, which interacts with Asp171. Second, although 4MCHA clearly forms a CH-π interaction, the phenyl ring of compound 1 is not completely stacked with the aromatic ring of Tyr237, which leads to a weaker π–π interaction. However, we considered compound 1 to be an acceptable starting molecule for optimization because, in many cases of high-throughput screening (HTS) campaigns, the IC_50_ of hit compounds exceed 10 μM^40^. In addition, the confirmation rate, which is the ratio of the number of compounds with confirmed enzymatic activity to the number of docking hits, increased to 2.27% because docking simulations were performed; for comparison, the hit rate for HTS is generally <1%^27^. Moreover, compound 1 is more drug-like than 4MCHA due to the fact that the quantitative estimate of its drug-likeness (QED)^41^ is greater than that of 4MCHA (compound 1 QED = 0.63 > 4MCHA = 0.47).

According to our FMO analysis results, compound 1 interacts with Gln64, Tyr73, Asp171, Gln201 and Tyr237. Tyr237 interacts with the aromatic ring of compound 1 through a π-π interaction, and Asp171 interacts with the amino group of compound 1 through a salt bridge with the strongest interaction energy (−16.18 kcal/mol). Asp171 belongs to the gatekeeping loop, which is important for substrate binding in the SpdSyn active site^28^. In the protein structure that we used for the docking simulation (PDB ID code: 3BWC), the gatekeeping loop is disordered because the structure was determined in substrate-free form. However, the gatekeeping loop is found to be ordered in the TcSpdSyn-compound 1 complex structure due to the attraction between Asp171 of this loop and compound 1. We also conducted MD simulations to analyze the interaction modes using compounds 1–4 and 4MCHA. The structures of the TcSpdSyn complex with compounds 2–4 were provided by docking simulations of these compounds using a TcSpdSyn-compound 1 X-ray structure with an ordered gatekeeping loop. The results obtained from MD simulation indicated that, unlike 4MCHA, each compound interacts with Asp171, Gln22, Gln64, Asp168, Gln201, Ser204 and Gly243. In contrast, S-adenosyl-3-thio-1,8-diaminooctane (AdoDATO, PDBID: 2I7C) and 5-(1H-benzimidazol-2-yl)pentan-1-amine (BIPA, PDBID: 4CWA), which are inhibitors of *P*. *falciparum* SpdSyn, they bind to both putrescine and dcSAM binding sites in reported co-crystal structures^42, 43^. Although, compounds 1, 3 and 4 have an amino-alkyl chain like BIPA their amino group should interact with Asp171 through a salt bridge with compound 1 bound to putrescine binding site while compounds 3 and 4 bound to the dcSAM site.

Our results suggest that the docking simulation is a useful method for discovering drug-like inhibitors of TcSpdSyn. Accordingly, we hope to demonstrate that such simulations represent a valuable tool for finding protein inhibitors in general and that the results obtained in this study could facilitate the development of TcSpdSyn-targeted anti-Chagas drugs.

## Methods

### Target identification

We used the iNTRODB database, which integrates information on the genes, proteins, and compounds related to the three species of parasitic *Trypanosoma*, to efficiently narrow the range of candidate target molecules^11^. Using iNTRODB, we identified four candidate target proteins from 9,078 genes using the two-step process shown in Supplementary Figure 1. The present study focuses on SpdSyn.

### Protein preparation and docking simulation

The TcSpdSyn structure of 3BWC, which was used as a docking target, was taken from PDB. This structure was subjected to hydrogenation to remove water molecules and optimize the energy by Maestro using the OPLS2005 force field^44^. Docking simulations were conducted for the putrescine-binding site of the prepared TcSpdSyn structure in the absence of putrescine substrate. While calculating the docking simulation, a grid box with dimensions of 20 × 20 × 20 Å^3^ was generated to maintain the TcSpdSyn putrescine-binding site. In addition, dcSAM was included as a SpdSyn cofactor.

We used the Glide standard-precision mode^45^ for docking simulations, and approximately 4,800,000 drug-like compounds, suitable for Lipinski’s rule of five^46^, from the Namiki Shoji Co., Ltd. (Tokyo, Japan) and Astellas Pharma Inc. (Ibaraki, Japan) in-house libraries were included in the simulation. The mapping table between the compound ID (denoted as supplier ID) and the compounds listed in Table 1 is shown in Supplementary Table 1. All calculations were performed using an HP Proliant SL390s G7 server with an Intel Xeon X5670 2.93 GHz core and five nodes in the TSUBAME2.5 supercomputer located at the Tokyo Institute of Technology.

### TcSpdSyn inhibition enzyme assay

The TcSpdSyn inhibition assay was performed essentially as previously^27^ described. Briefly, the assay was an enzyme-coupled assay incorporating spermidine/spermine N(1)-acetyltransferase 1 (SSAT1). 7-Diethylamino-3-(4-maleimidylphenyl)-4-methylcoumarin (CPM) (cat. D-346, Thermo Fischer Scientific) was used to measure the coenzyme A produced by the SSAT1 reaction. The reaction mixture contained 50 mM 4-(2-hydroxyethyl)-1-piperazineethanesulfonic acid (HEPES) buffer (pH 7.5), 10 μM ethylenediaminetetraacetic acid (EDTA), 0.01% Tween 20, 14.7 nM TcSpdSyn, 50 μM dcSAM, 50 μM putrescine, 15 μM Ac-CoA (acetyl-coenzyme), and 0.83 nM SSAT1 in the presence or absence of the compounds and was incubated at room temperature for 30 min. The concentrations of putrescine and dcSAM were adjusted according to their *K*_*m*_ values (data not shown). The fluorescence signal was then detected in a plate reader (Paradigm, Molecular Devices Inc.) with excitation at 405 nm and emission at 530 nm. Each IC_50_ value was measured using eight different concentrations in quadruplicate. The dose-response curves of the compounds in Table 1 are shown in Supplementary Figure 3. Compounds 1, 3, and 4 were used in their salt forms, whereas compound 2 was used in its free form. The corresponding salts were oxalic acid for compound 1 and HCl for compounds 3 and 4. These compounds were dissolved in dimethyl sulfoxide (DMSO), and the maximum final DMSO concentration in the assays was 1.3%.

### Crystallization, data collection, and refinement

X-ray crystallography was carried out essentially as previously described^27^. Co-crystals of TcSpdSyn in complex with dcSAM and compound 1 were obtained using a sitting-drop vapor diffusion method. Before crystallization, 15 mg/mL TcSpdSyn was mixed with dcSAM and compound 1 at final concentrations of 2 and 5 mM, respectively. The reservoir solution consisted of 100 mM bis-Tris (pH 5.5–6.5), 200 mM ammonium sulfate, and 10–15% (*w/v*) polyethylene glycol with a molecular weight of 4000 g/mol (PEG 4000). The crystals were transferred into a mother liquor containing 20% (*v/v*) glycerol as a cryoprotectant and flash frozen in liquid nitrogen. X-ray diffraction data were collected at the Photon Factory AR-NE3A beamline using the robotic sample changer and the automated data collection system^47, 48^. The structure was solved by molecular replacement with Phaser^49^ using the apo-structure of TcSpdSyn (PDB ID code: 3BWB) as a search model. After structural refinement using REFMAC^50^, electron density maps corresponding to dcSAM and compound 1 were clearly obtained and fitted using AFITT (OpenEye Scientific). The final structure was deposited in PDB (PDB ID code: 5B1S).

### Interaction energy analysis for SpdSyn–ligand complex

FMO calculation input files were generated using FMOutil Version 2.1(https://staff.aist.go.jp/d.g.fedorov/fmo/fmoutil.html), and calculations were performed for each A-chain monomer using GAMESS^51^ at the MP2/6–31 G level. Interaction energy analysis was performed using the analytical tool Facio^52^ on the basis of pair interaction energy decomposition analysis, as proposed by Fedorov *et al*.^53^.

### MD simulation

Compounds 1–4 and 4MCHA were used in the MD simulations. Complexes of TcSpdSyn and compounds 2–4 were generated by docking simulations using the TcSpdSyn-compound 1 complex structure similarly described at protein preparation and docking simulation section. For compound 1 and 4MCHA, the obtained crystal structures were used in the MD simulation. The simulation system was prepared using Desmond Version 3.5 with default settings. The temperature and pressure of the system were set at 300 K and 1 atm, respectively. The time step and structure sampling intervals were set at 2 fs and 1 ps, respectively. We conducted the simulation under the isothermal-isobaric (NPT) ensemble for 20 ns.

## Electronic supplementary material

Supplementary Information
