# Supplementary material for: In silico, in vitro, X-ray crystallography, and integrated strategies for discovering spermidine synthase inhibitors for Chagas disease
Source: Sci Rep. 2017 Jul 27;7:6666. doi: 10.1038/s41598-017-06411-9 (PMC5532286; doi:10.1038/s41598-017-06411-9)
Supplement: Supplementary file 1 — Supplementary Information [file 41598_2017_6411_MOESM1_ESM.pdf]

**Supplementary Materials for**

# *In silico*, *in vitro*, X-ray crystallography, and integrated strategies for discovering spermidine synthase inhibitors for Chagas disease

**Authors**

Ryunosuke Yoshino[3,4,6], Nobuaki Yasuo[2,6], Yohsuke Hagiwara[2,5], Takashi Ishida[1,2,6], Daniel Ken Inaoka[7,8], Yasushi Amano[5], Yukihiro Tateishi[5], Kazuki Ohno[2,5,9], Ichiji Namatame[5], Tatsuya Niimi[5], Masaya Orita[5], Kiyoshi Kita[7,8], Yutaka Akiyama[1,2,6], and Masakazu Sekijima[1,2,3,6*]

**Affiliations**

[1]Advanced Drug Discovery Unit, Institute of Innovative Research, Tokyo Institute of Technology, 4259-J3-23, Nagatsuta-cho, Midori-ku, Yokohama, 226-8502, JAPAN

[2]Education Academy of Computational Life Sciences (ACLS), Tokyo Institute of Technology, Yokohama 226-8501 Japan

[3]Global Scientific Information and Computing Center, Tokyo Institute of Technology, 2-12-1 Ookayama, Meguro-ku, Tokyo 152-8550 Japan

[4]Graduate School of Agricultural and Life Sciences, The University of Tokyo, Bunkyo-ku, Tokyo 113-8657 Japan

[5]Medicinal Chemistry Research Labs, Drug Discovery Research, Astellas Pharma Inc, 21 Miyukigaoka, Tsukuba, Ibaraki 305-8585 Japan

[6]Department of Computer Science, Graduate School of Information Science and Engineering, Tokyo Institute of Technology, Meguro-ku, Tokyo 152-8550, Japan

[7]Department of Biomedical Chemistry, Graduate School of Medicine, The University of Tokyo, Bunkyo-ku, Tokyo, 113–0033, Japan

[8]School of Tropical Medicine and Global Health, Nagasaki University, Sakamoto,

Nagasaki, 852–8523, Japan

[9]Present affiliation: Catalyst Inc., Risona Kudan Building 5F KS Floor, 1－5－6 Kudan Minami, Chiyoda-ku, Tokyo 102-0074 Japan

Correspondence to sekijima@c.titech.ac.jp

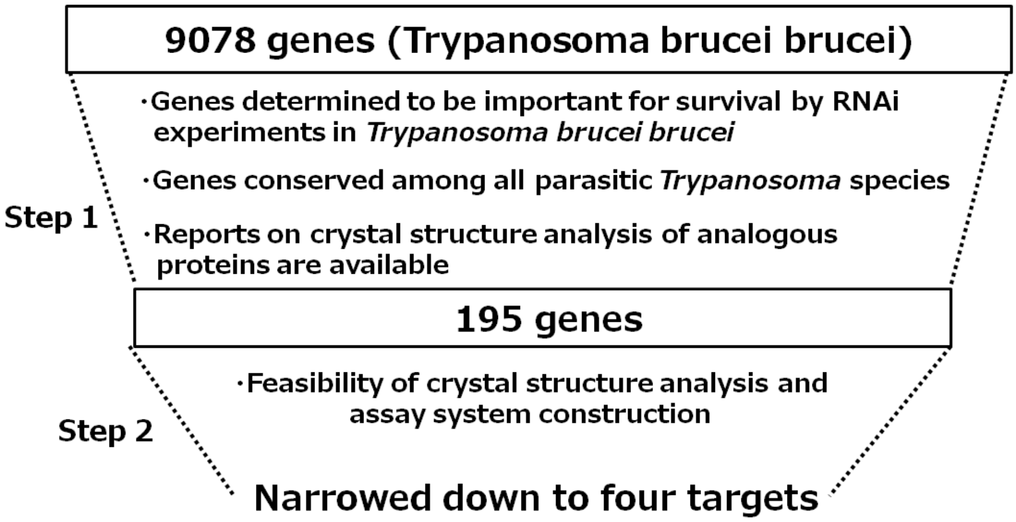


**Supplementary Figure 1.** Search method for target proteins using iNTRODB.

a

```
>Tb09.v1.0380 |||spermidine synthase, putative|Trypanosoma
          brucei|chr 9|||Manual
         Length = 298

 Score =  431 bits (1107), Expect = e-154,   Method: Compositional matrix adjust.
 Identities = 199/296 (67%), Positives = 239/296 (80%), Gaps = 1/296 (0%)

Query: 1   MPGSELISGGWFREENDQWPGQAMSLRVEKVLYDAPTKFQHLTIFESDPKGPWGTVMALD 60
           MPG  L++ GWFREEN QWPGQAMS +VE+VL+D PTKFQHL+IFE+DPKGPWGTVM LD
Sbjct: 1   MPGPGLLADGWFREENGQWPGQAMSFKVEEVLHDTPTKFQHLSIFETDPKGPWGTVMTLD 60

Query: 61  GCIQVTDYDEFVYHEVLGHTSLCSHPKPXXXXXXXXXXXXXXXXXXXHGTVEHCDLVDID 120
           GCIQ+TD+DEFVYHE+L HT LC+HP P                   HGTV+ C LVDID
Sbjct: 61  GCIQLTDFDEFVYHEMLSHTPLCAHPDPVDVLIIGGGDGGVMREVLRHGTVKRCVLVDID 120

Query: 121 GEVMEQSKQHFPQISRSLTDPRATVRVDDGLAFVRQTPDNTYDVVIIDTTDPAGPASELF 180
           G+V+E SK++FPQIS   +DPRA VRV DG+AFVR+    ++DVVIIDTTDP GPA+ELF
Sbjct: 121 GDVIEASKKYFPQISSGFSDPRADVRVGDGVAFVREAASESFDVVIIDTTDPDGPAAELF 180

Query: 181 GEAFYKHVLRILKPDGICCNQGESIWLNLELIEKMSRFIR-ETGFASVQYALMHVPTYPC 239
           GE FY+ VLRILKP GICCNQGES+WLN  LIE M+ FI+ + GFASV+YA+++ PTYPC
Sbjct: 181 GEKFYRDVLRILKPRGICCNQGESVWLNRNLIEGMADFIKNKVGFASVKYAMIYTPTYPC 240

Query: 240 GSIGTLICSKKAGVDVTKPLRPVEDMPFAKDLKYYDSEMHKASFALPRFARHINNS 295
           GSIG+LICSK AGVDVT+P+RPVE MPFA +LKYYDS++HKA+F LPRFARH+N +
Sbjct: 241 GSIGSLICSKVAGVDVTQPVRPVESMPFAGELKYYDSDVHKAAFVLPRFARHLNQN 296
```

b

```
>LmjF.04.0580 | organism=Leishmania_major_strain_Friedlin |
          product=spermidine synthase |
          location=LmjF.04:239178-240080(-) | length=300
         Length = 300

 Score =  380 bits (977), Expect = e-134,   Method: Compositional matrix adjust.
 Identities = 180/299 (60%), Positives = 218/299 (72%), Gaps = 6/299 (2%)

Query: 1   MPGSELISGGWFREENDQWPGQAMSLRVEKVLYDAPTKFQHLTIFESDPKGPWGTVMALD 60
           MPG  L+  GWFREE+  WPGQA  L+VEKVLYD PT+FQHLT+FESDP GPWGTVM LD
Sbjct: 1   MPGPGLLPDGWFREESTMWPGQAQGLKVEKVLYDQPTEFQHLTVFESDPSGPWGTVMTLD 60

Query: 61  GCIQVTDYDEFVYHEVLGHTSLCSHPKPXXXXXXXXXXXXXXXXXXXH-----GTVEHCD 115
           G IQ+TDYDEFVYHE+L + SL  H KP                   H     G V+  +
Sbjct: 61  GAIQLTDYDEFVYHEMLANLSLACHHKPERVLVIGGGDGGVVREVLRHKSEKDGVVQSVE 120

Query: 116 LVDIDGEVMEQSKQHFPQISRSLTDPRATVRVDDGLAFVRQTPDNTYDVVIIDTTDPAGP 175
           LVDIDG VM+QSK+HFPQ++    +P  T  V DG AFVR  PD+ YDV+IIDTTDP GP
Sbjct: 121 LVDIDGAVMQQSKKHFPQVACGFANPCVTATVGDGAAFVRNVPDSVYDVIIIDTTDPKGP 180

Query: 176 ASELFGEAFYKHVLRILKPDGICCNQGESIWLNLELIEKMSRFI-RETGFASVQYALMHV 234
           ASELFG  FY +VLRIL+P G+ CNQGES+WL+  LIEKM  F+ ++ GFA+V+YA++++
Sbjct: 181 ASELFGADFYTNVLRILRPGGVVCNQGESVWLHRPLIEKMMGFLKKDIGFATVKYAMIYI 240

Query: 235 PTYPCGSIGTLICSKKAGVDVTKPLRPVEDMPFAKDLKYYDSEMHKASFALPRFARHIN 293
           PTYPCGSIGTL+C+K A  DVT P+RPVE + FA  LKYY S+MHKA+F LPRFA H+N
Sbjct: 241 PTYPCGSIGTLVCAKSADTDVTVPMRPVESLGFADQLKYYSSDMHKAAFVLPRFAAHLN 299
```

c

```
>gi|63253298|ref|NP_003123.2| spermidine synthase [Homo sapiens]
         Length = 302

 Score =  224 bits (571), Expect = 5e-72,   Method: Compositional matrix adjust.
 Identities = 114/293 (38%), Positives = 172/293 (58%), Gaps = 6/293 (2%)

Query: 3   GSELISGGWFREENDQWPGQAMSLRVEKVLYDAPTKFQHLTIFESDPKGPWGTVMALDGC 62
           G   I  GWFRE    WPGQA+SL+VE++L+   +++Q + +F S     +G V+ LDG
Sbjct: 12  GPAAIREGWFRETCSLWPGQALSLQVEQLLHHRRSRYQDILVFRSKT---YGNVLVLDGV 68

Query: 63  IQVTDYDEFVYHEVLGHTSLCSHPKPXXXXXXXXXXXXXXXXXXXHGTVEHCDLVDIDGE 122
           IQ T+ DEF Y E++ +  LCSHP P                   H +VE     +ID +
Sbjct: 69  IQCTERDEFSYQEMIANLPLCSHPNPRKVLIIGGGDGGVLREVVKHPSVESVVQCEIDED 128

Query: 123 VMEQSKQHFPQISRSLTDPRATVRVDDGLAFVRQTPDNTYDVVIIDTTDPAGPASELFGE 182
           V++ SK+  P ++   +  + T+ V DG  F++Q  D  +DV+I D++DP GPA  LF E
Sbjct: 129 VIQVSKKFLPGMAIGYSSSKLTLHVGDGFEFMKQNQD-AFDVIITDSSDPMGPAESLFKE 187

Query: 183 AFYKHVLRILKPDGICCNQGESIWLNLELIEKMSRFIRETGFASVQYALMHVPTYPCGSI 242
           ++Y+ +   LK DG+ C QGE  WL+L+LI++M +F +   F  V YA   +PTYP G I
Sbjct: 188 SYYQLMKTALKEDGVLCCQGECQWLHLDLIKEMRQFCQSL-FPVVAYAYCTIPTYPSGQI 246

Query: 243 GTLICSKKAGVDVTKPLRPVEDMPFAK-DLKYYDSEMHKASFALPRFARHINN 294
           G ++CSK    +  +P++P+     A+  LKYY+S++H+A+F LP FAR   N
Sbjct: 247 GFMLCSKNPSTNFQEPVQPLTQQQVAQMQLKYYNSDVHRAAFVLPEFARKALN 299
```

**Supplementary Figure 2.** Aligned protein sequence of spermidine synthase between *Trypanosoma cruzi* (CL Brener strain) and A: *T. brucei*, B: *Leishmania major*, C: *Homo sapiens*. Aligned sequences were obtained from iNTRODB.

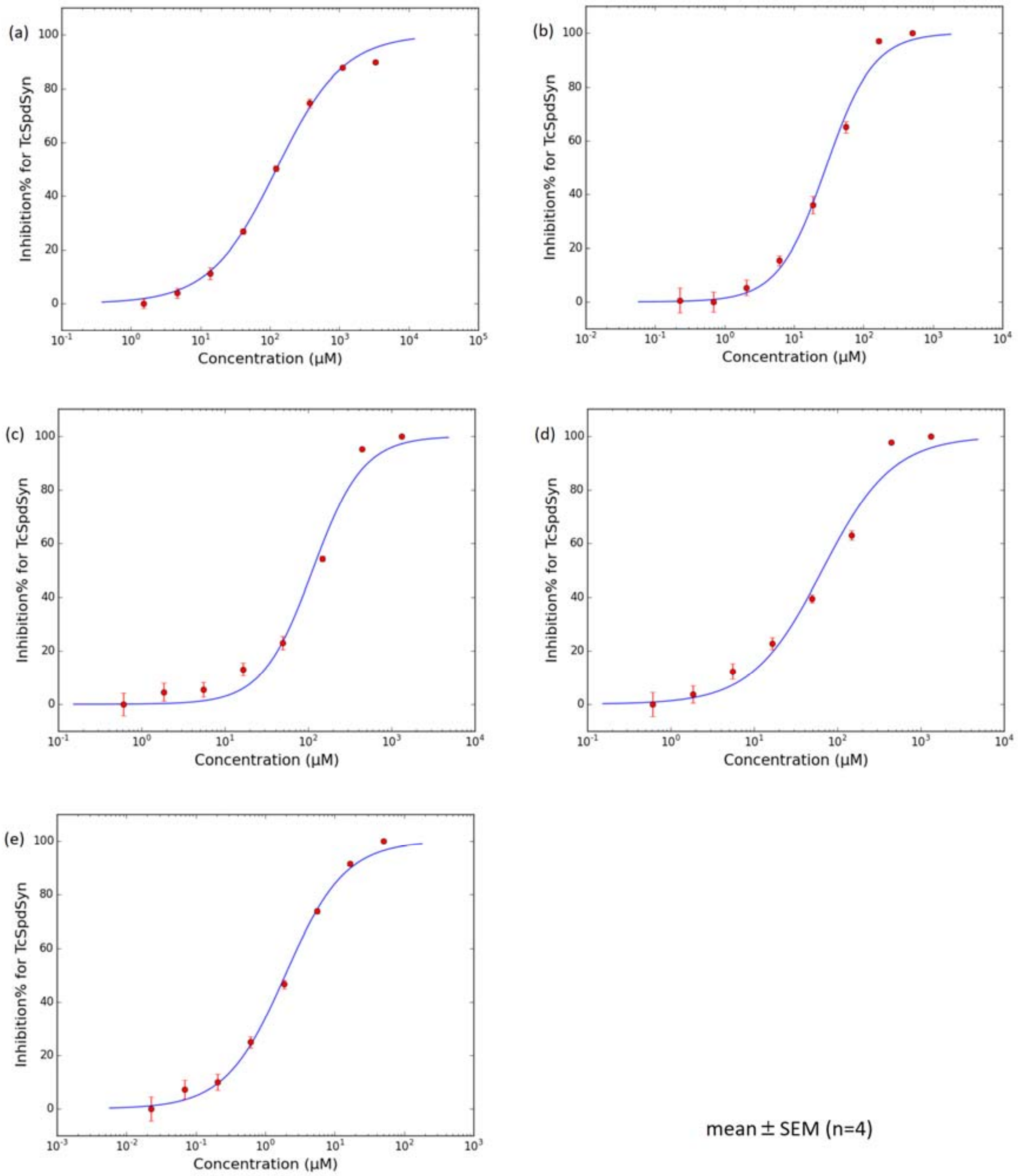


**Supplementary Figure 3.** The fitting curve for $IC_{50}$ calculation of the compounds on Table 1 (Compounds 1, 2, 3, 4, and 4MCHA correspond to (a), (b), (c), (d) and (e), respectively). The vertical line and horizontal axis's represent inhibition% for TcSpdSyn and compound concentration. The closed circles show the inhibition% measured at each dose. The error bars indicate the standard error of the inhibition% measured at each dose. For curve fitting, the following four paramter logoistic (4PL) model[1] is used. The paramter values of the equation of each compound are shown in Supplementary Table 3.

$$y = min + \frac{max - min}{1 + 10^{(Hill(Log10(X50) - x))}}$$

**Supplementary Table 1.** Supplier ID and Namiki ID of the active compounds by *in vitro* assay listed in Table 1.

| Compound ID | Supplier Name | Supplier ID | Namiki ID |
|---|---|---|---|
| 1 | Bepharm | B21234 | NS-00034421 |
| 2 | Vitas-M | STK107845 | NS-00694195 |
| 3 | Pharmeks | P2001S-338404 | NS-03964486 |
| 4 | Enamine | EN300-115921 | NS-00027256 |

**Supplementary Table 2.** Supplier ID and Namiki ID of inactive compounds by *in vitro* assay.

| Namiki ID | Supplier ID | Supplier Name |
|---|---|---|
| NS-01988866 | IBS-L0127228 | Alinda |
| NS-04133406 | AC-20091 | APIChem |
| NS-00084264 | OR13234 | Apollo |
| NS-08456239 | ADM 12409198 | Asinex |
| NS-09711136 | ART 09584052 | Asinex |
| NS-09686125 | ART 12883679 | Asinex |
| NS-09723909 | ART 15417152 | Asinex |
| NS-00738895 | ASN 03204271 | Asinex |
| NS-00553327 | BAS 00112672 | Asinex |
| NS-00568801 | BAS 00132206 | Asinex |
| NS-00557872 | BAS 00162617 | Asinex |
| NS-00375233 | BAS 00485302 | Asinex |
| NS-00456710 | BAS 01052336 | Asinex |
| NS-00683031 | BAS 03673624 | Asinex |
| NS-06088261 | B10057 | Bepharm |
| NS-01984066 | B110683 | Bepharm |
| NS-01984075 | B11935 | Bepharm |
| NS-00242318 | B126389 | Bepharm |
| NS-00216529 | B185962 | Bepharm |
| NS-00242327 | B19373 | Bepharm |
| NS-08726051 | B225643 | Bepharm |

| | | |
|---|---|---|
| NS-00502269 | B226825 | Bepharm |
| NS-00084817 | B237070 | Bepharm |
| NS-00520067 | B34480 | Bepharm |
| NS-01447165 | B53509 | Bepharm |
| NS-00038373 | B55162 | Bepharm |
| NS-00027436 | B6027 | Bepharm |
| NS-00026629 | B7480 | Bepharm |
| NS-00086239 | B7776 | Bepharm |
| NS-00106974 | B83970 | Bepharm |
| NS-00240797 | B86318 | Bepharm |
| NS-00042374 | B94867 | Bepharm |
| NS-04691291 | 2F-088 | Bionet |
| NS-00897223 | 1C-097 | Bionet(Fragment) |
| NS-01971130 | IN-0425 | CombiBlocks |
| NS-00045707 | QA-1027 | CombiBlocks |
| NS-00210693 | QA-3805 | CombiBlocks |
| NS-02726054 | QC-6510 | CombiBlocks |
| NS-03104067 | QE-9891 | CombiBlocks |
| NS-00025515 | QY-1767 | CombiBlocks |
| NS-00096534 | SS-4524 | CombiBlocks |
| NS-00422542 | EN300-112596 | Enamine |
| NS-03042830 | EN300-113084 | Enamine |
| NS-00024529 | EN300-122589 | Enamine |
| NS-10494236 | EN300-127485 | Enamine |
| NS-00135682 | EN300-13617 | Enamine |
| NS-00021451 | EN300-13896 | Enamine |
| NS-03065527 | EN300-139143 | Enamine |
| NS-00025508 | EN300-15718 | Enamine |
| NS-00044917 | EN300-160189 | Enamine |
| NS-00034115 | EN300-175002 | Enamine |
| NS-00025550 | EN300-18624 | Enamine |
| NS-00096048 | EN300-20404 | Enamine |

| | | |
|---|---|---|
| NS-00095940 | EN300-215558 | Enamine |
| NS-00044424 | EN300-31275 | Enamine |
| NS-05620599 | EN300-33359 | Enamine |
| NS-00208253 | EN300-34367 | Enamine |
| NS-00040634 | EN300-36560 | Enamine |
| NS-00039012 | EN300-38966 | Enamine |
| NS-00083514 | EN300-56240 | Enamine |
| NS-00127946 | EN300-61777 | Enamine |
| NS-07300875 | EN300-64804 | Enamine |
| NS-04286829 | EN300-68454 | Enamine |
| NS-01820579 | EN300-69071 | Enamine |
| NS-05682291 | EN300-82463 | Enamine |
| NS-00000321 | Z104507050 | Enamine |
| NS-09460347 | Z1123821842 | Enamine |
| NS-09851924 | Z1130245470 | Enamine |
| NS-05677846 | Z1198335676 | Enamine |
| NS-02654486 | Z1255356155 | Enamine |
| NS-10120169 | Z169504308 | Enamine |
| NS-03448452 | Z237732596 | Enamine |
| NS-00652324 | Z238921002 | Enamine |
| NS-01767178 | Z31375831 | Enamine |
| NS-01639043 | Z31380603 | Enamine |
| NS-02048933 | Z370706740 | Enamine |
| NS-08389397 | Z384973502 | Enamine |
| NS-00036560 | Z415740552 | Enamine |
| NS-04286824 | Z432090862 | Enamine |
| NS-08436212 | Z445132884 | Enamine |
| NS-01248894 | Z45415551 | Enamine |
| NS-00250557 | Z56780034 | Enamine |
| NS-06342424 | Z600584194 | Enamine |
| NS-07238675 | Z649635088 | Enamine |
| NS-00206553 | Z90660992 | Enamine |

| | | |
|---|---|---|
| NS-00029324 | Z944939438 | Enamine |
| NS-03050150 | 12R0026 | JWPharmlab |
| NS-03052261 | 20R0212 | JWPharmlab |
| NS-01958208 | LT00007983 | Labotest |
| NS-05078226 | LT00030844 | Labotest |
| NS-01963106 | LT00050116 | Labotest |
| NS-01971832 | LT00118654 | Labotest |
| NS-01976901 | LT00154482 | Labotest |
| NS-01981402 | LT-IV X 478 | Labotest |
| NS-02049344 | F1278-0327 | LifeChemicals |
| NS-04370463 | F3366-5220 | LifeChemicals |
| NS-00021546 | 9899 | Matrix |
| NS-00024028 | 10868 | Matrix |
| NS-00022663 | 18523 | Matrix |
| NS-03120259 | 21508 | Matrix |
| NS-00096533 | 33847 | Matrix |
| NS-06850867 | 41077 | Matrix |
| NS-00375649 | BTB12148 | Maybridge |
| NS-00625817 | CD07798 | Maybridge |
| NS-00041234 | KM01833 | Maybridge |
| NS-02168278 | KM09877 | Maybridge |
| NS-02171378 | RDP00177 | Maybridge |
| NS-02172479 | RF00498 | Maybridge |
| NS-02172984 | RF02876 | Maybridge |
| NS-00042069 | RJC00810 | Maybridge |
| NS-02181735 | SEW03911 | Maybridge |
| NS-00101613 | SPB07361 | Maybridge |
| NS-04428093 | CC75719 | Maybridge(Fragment) |
| NS-02142145 | 13321 | MolMall |
| NS-02142989 | 15379 | MolMall |
| NS-02143192 | 15633 | MolMall |
| NS-02143187 | 102931 | MolMall |

| | | |
|---|---|---|
| NS-04364531 | 1049802 | OTAVA |
| NS-05686762 | 1192847 | OTAVA |
| NS-04409108 | 7020692941 | OTAVA |
| NS-00601376 | P2000S-19366 | Pharmeks |
| NS-00616224 | P2000S-23760 | Pharmeks |
| NS-00599907 | P2000S-26470 | Pharmeks |
| NS-00629207 | P2000S-27173 | Pharmeks |
| NS-02281209 | P2001S-052746 | Pharmeks |
| NS-00638619 | P2001S-063604 | Pharmeks |
| NS-02293313 | P2001S-113312 | Pharmeks |
| NS-01448839 | P2001S-160661 | Pharmeks |
| NS-00694795 | P2001S-168762 | Pharmeks |
| NS-02320185 | P2001S-186681 | Pharmeks |
| NS-02320927 | P2001S-187781 | Pharmeks |
| NS-00881867 | P2001S-238236 | Pharmeks |
| NS-01061606 | P2001S-264815 | Pharmeks |
| NS-02234815 | P2000N-10331 | Pharmeks(Natural) |
| NS-02235337 | P2000N-11786 | Pharmeks(Natural) |
| NS-02250802 | P2000N-37233 | Pharmeks(Natural) |
| NS-02503810 | OSSK_485183 | Princeton |
| NS-09668660 | OSSL_477426 | Princeton |
| NS-01484266 | OSSL_822359 | Princeton |
| NS-02648504 | PH012669 | SALOR |
| NS-00475129 | R295736 | SALOR |
| NS-02628888 | R436860 | SALOR |
| NS-02642429 | S136107 | SALOR |
| NS-04101213 | S914789 | SALOR |
| NS-00242430 | 1-0026 | ScientificExchange |
| NS-03323092 | Z-007027 | ScientificExchange |
| NS-02719860 | Z-025192 | ScientificExchange |
| NS-02683904 | AJ-333/15478078 | SPECS |
| NS-02721148 | ST002006 | TimTec |

| | | |
|---|---|---|
| NS-00690438 | STK016799 | Vitas-M |
| NS-02525824 | STK163122 | Vitas-M |
| NS-02596772 | STK220715 | Vitas-M |
| NS-03444130 | STK280472 | Vitas-M |
| NS-05176117 | STK298937 | Vitas-M |
| NS-05615458 | STK338050 | Vitas-M |
| NS-01447216 | STK368001 | Vitas-M |
| NS-03667421 | STK387017 | Vitas-M |
| NS-06400628 | STK521785 | Vitas-M |
| NS-00472084 | STK734312 | Vitas-M |
| NS-00873275 | STL032034 | Vitas-M |
| NS-01963305 | STL033042 | Vitas-M |
| NS-00671753 | STL327502 | Vitas-M |
| NS-00469312 | UZI/2599943 | Zelinsky |
| NS-00505849 | UZI/2625541 | Zelinsky |
| NS-02729450 | UZI/2647801 | Zelinsky |
| NS-03081113 | UZI/2655098 | Zelinsky |
| NS-00354396 | UZI/6095081 | Zelinsky |
| NS-01451287 | UZI/6188274 | Zelinsky |
| NS-00464302 | UZI/7131862 | Zelinsky |
| NS-01036208 | UZI/8190366 | Zelinsky |
| NS-00240683 | UZI/9019794 | Zelinsky |
| NS-10533350 | UZI/9062663 | Zelinsky |

Supplementary Table 3. The parameter values of the equation used for the curve fitting.

| Compound ID | min | Max | Hill | LoggedX50 |
|---|---|---|---|---|
| 1 | 0.00 | 100.00 | 0.90 | 2.09 |
| 2 | 0.00 | 100.00 | 1.27 | 1.46 |
| 3 | 0.00 | 100.00 | 1.44 | 2.05 |
| 4 | 0.00 | 100.00 | 1.03 | 1.82 |
| 4MCHA | 0.00 | 100.00 | 1.01 | 0.29 |

## References


1. Fomenko, I, Durst, M & Balaban, D. Robust regression for high throughput drug screening. *Comput Meth Programs Biomed* **82**, 31-37 (2006).
